# A New Principle in the Application of the Obstetric Forceps

**Published:** 1876-11

**Authors:** Samuel D. Turney

**Affiliations:** Circleville, Ohio, Adjunct Professor of Obstetrics in Starling Medical College, Columbus, Ohio


### A NEW PRINCIPLE IN THE APPLICATION OF THE OBSTETRIC FORCEPS.

By SAMUEL D. TURNEY, of Circleville, Ohio,
Adjunct Professor of Obstetrics in Starling Medical College, Columbus, Ohio.
(*Read before the Central Ohio Medical Association.*)

It has been but a few years since all medical authorities agreed in advising the use of the obstetrical forceps only when the life of the child or of the mother was seriously endangered. Acting upon this teaching, the woman was often left in the second stage of labor until on the verge of exhaustion, and only when death seemed inevitable without instrumental interference, the attending physician reluctantly applied the forceps. From the long-continued pressure of the head of the child, and the disturbance of circulation incident to these protracted labors, followed a train of evils—fistulæ, cellulitis, peritonitis, and abscesses—which, on the *post hoc propter hoc* theory, were illogically laid to the account of the instruments.

A better acquaintance with the true cause of these diseased conditions has at length exonerated the forceps from these unjust charges, and the physician of to-day knows that in their timely and skilful use lies safety for the mother and child.

But although fairly exonerated from responsibility for most of the diseases which follow child-bearing, there is one injury which very frequently accompanies, and which is undoubtedly often due to, forceps delivery. This is laceration of the perineum. Even when the greatest care is exercised to avoid injurious haste, and to make traction only in the line of the curvature of the inferior strait of the pelvis, laceration of the perineum is, I think all physicians will admit, very common in cases terminated by such instrumental means.

Is this accident unavoidable? Is it justly chargeable to the use of forceps, or is it due to a faulty mode of using them?

To consider these questions, let us briefly look at the mechanism of the passage of the head of the child through the vulva. In unaided natural delivery, toward the termination of the second stage of labor, with each contraction of the uterus the head presses upon and distends the perineum; as the pain subsides the head recedes, and during the interval of repose, the perineum, freed from distending force, recovers a portion of its elasticity and partially recontracts. This process of alternate distention and contraction goes on for a longer or shorter period, until the perineum, losing rigidity, allows of the full dilatation of the vulva, and the child is born; or the perineum remaining rigid, the expulsive efforts becoming progressively more powerful, it gives way, and we have some form of laceration. In these cases of natural delivery there is a disproportion between the expulsive force and the dilatability of the perineum, and whatever increases that force in this condition of the perineum increases the danger of its laceration.

The ordinary mode of using the obstetrical forceps, when the directions laid down in systematic works on obstetrics are followed, does this, and is, consequently, a very frequent cause of this injury. The universal teaching, so far as I know, in the use of these instruments is, if there are uterine pains, to make traction during the pain—that is during the expulsive effort—this rule, probably all right when the head is in the cavity of the pelvis, is, I think, all wrong when the head is at the outlet.

This increase of expulsive force is, I believe, a capital error, and a source of much mischief. Believing this, I have for some years adopted an entirely different mode, making little or no traction during the pain, but in the interval of the pain using enough traction to prevent recession of the head of the child, and holding it firmly against the perineum. This prevents a recontraction of the perineum, and converts a violent intermittent distensive force into one which is slowly acting and persistent. Under this moderate but constant pressure the perineum surely and safely dilates, and laceration is avoided.

I have been satisfied with the results of this practice, and think the rule in the use of the forceps, when the head is pressing upon the perineum, should be reversed, and should stand: *Make no traction during the pain ; let the traction be made in the interval of pain.—Ohio Med. & Surg. Jour.*

